# 4-(2,3,4-Trimeth­oxy-6-methyl­benzyl­idene­amino)phenol

**DOI:** 10.1107/S1600536809008046

**Published:** 2009-03-11

**Authors:** Cheng-Yun Wang

**Affiliations:** a Department of Chemistry and Chemical Engineering, Weifang University, Weifang 261061, People’s Republic of China

## Abstract

The asymmetric unit of the title compound, C_17_H_19_NO_4_, contains two independent mol­ecules in which the dihedral angles between the two benzene rings are 83.1 (2) and 88.5 (2)°. Each mol­ecule adopts a *trans* configuration with respect to the C=N bond. In the crystal structure, mol­ecules are linked by inter­molecular O—H⋯N hydrogen bonds, forming two independent one-dimensional chains running along the *b*-axis direction.

## Related literature

For the preparation, properties and applications of Schiff bases, see: Yu *et al.* (2007 ▶). For a related structure, see: Wang (2009 ▶).
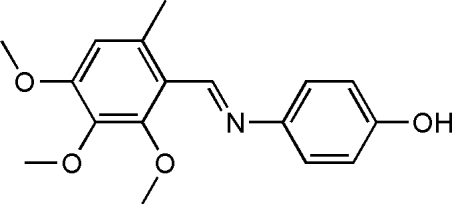


## Experimental

### 

#### Crystal data

C_17_H_19_NO_4_
*M* *_r_* = 301.33
Orthorhombic, 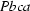

*a* = 20.045 (2) Å
*b* = 13.2042 (19) Å
*c* = 24.253 (3) Å
*V* = 6419.2 (14) Å^3^
*Z* = 16
Mo *K*α radiation
μ = 0.09 mm^−1^
*T* = 298 K
0.49 × 0.48 × 0.42 mm

#### Data collection

Bruker SMART CCD diffractometer
Absorption correction: multi-scan (*SADABS*; Sheldrick, 1996 ▶) *T*_min_ = 0.958, *T*_max_ = 0.964
25460 measured reflections
5654 independent reflections
2589 reflections with *I* > 2σ(*I*)
*R*_int_ = 0.092

#### Refinement

*R*[*F* ^2^ > 2σ(*F* ^2^)] = 0.063
*wR*(*F* ^2^) = 0.206
*S* = 1.12
5654 reflections
405 parameters
H-atom parameters constrained
Δρ_max_ = 0.21 e Å^−3^
Δρ_min_ = −0.22 e Å^−3^

### 

Data collection: *SMART* (Bruker, 1997 ▶); cell refinement: *SAINT* (Bruker, 1997 ▶); data reduction: *SAINT*; program(s) used to solve structure: *SHELXS97* (Sheldrick, 2008 ▶); program(s) used to refine structure: *SHELXL97* (Sheldrick, 2008 ▶); molecular graphics: *SHELXTL* (Sheldrick, 2008 ▶) and *PLATON* (Spek, 2009 ▶); software used to prepare material for publication: *SHELXTL*.

## Supplementary Material

Crystal structure: contains datablocks global, I. DOI: 10.1107/S1600536809008046/lh2773sup1.cif

Structure factors: contains datablocks I. DOI: 10.1107/S1600536809008046/lh2773Isup2.hkl

Additional supplementary materials:  crystallographic information; 3D view; checkCIF report

## Figures and Tables

**Table 1:** Hydrogen-bond geometry (Å, °)

*D*—H⋯*A*	*D*—H	H⋯*A*	*D*⋯*A*	*D*—H⋯*A*
O4—H4⋯N1^i^	0.82	2.16	2.866 (5)	144
O8—H8⋯N2^ii^	0.82	1.99	2.777 (5)	161

Symmetry codes: (i) 
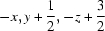
; (ii) 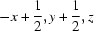.

**Fig. 1.**
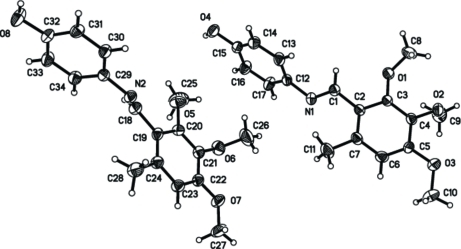
The asymmetric unit of (I), drawn with 30% probability ellipsoids.

**Fig. 2.**
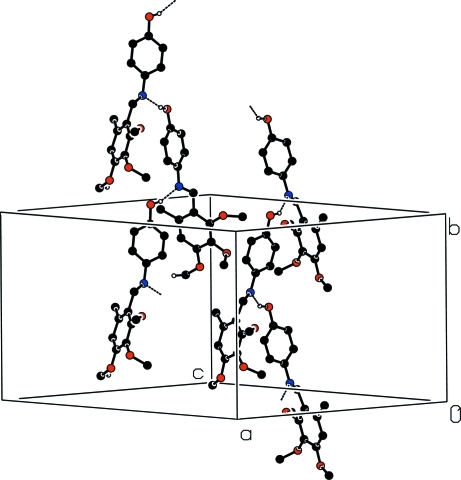
Part of the crystal structure of (I) with hydrogen bonds drawn as dashed lines.

## supplementary crystallographic information

### Comment

The preparation, properties and applications of Schiff bases are important in the development of coordination chemistry (see e.g. Yu *et al.*, 2007). In this paper, the structure of the title compound, (I), is reported. The asymmetric unit of (I) is shown in Fig. 1. The bond lengths and angles of the title compound agree with those in the related compound (*E*)—*N*-(2,3,4-Trimethoxy-6-methylbenzylidene)naphthalen-1-amine (Wang, 2008), as representative example. The asymmetric unit of the title compound consists of two independent molecules, in which the dihedral angles between the two benzene rings in each are 83.1 (2)° [for rings C2-C7 and C12-C17] and 88.5 (2)° [for rings C19-C24 and C29-C34]. The molecules adopt a *trans* configuration about the central C=N bond. In the crystal structure, molecules are linked by intermolecular O—H···N hydrogen bonds to form two independent one-dimensional chains running along the b axis direction (Fig. 2 and Table 1).

### Experimental

A mixture of 4-aminophenol (0.545 g, 5 mmol) and 2,3,4-trimethoxy-6-methylbenzaldehyde (1.04 g, 5 mmol) in ethyl alcohol (30 ml) was refluxed for 2 h. After cooling the precipitate was filtered and dried. The crude product of 20 mg was dissolved in 20 ml of ethyl alcohol by heating on a magnetic stirrer. The solution was filtered to remove impurities, and then left at room temperature. After a week single crystals of (I) suitable for structure determination were obtained.

### Refinement

The H atoms were positioned geometrically (C—H = 0.93–0.96 Å; O-H = 0.82Å) and refined as riding with *U*_iso_(H) = 1.2*U*_eq_(C) or 1.5 *U*_eq_(methyl C or O).

### Crystal data

**Table:**

C_17_H_19_NO_4_	*F*(000) = 2560
*M**_r_* = 301.33	*D*_x_ = 1.247 Mg m^−^^3^
Orthorhombic, *P**b**c**a*	Mo *K*α radiation, λ = 0.71073 Å
Hall symbol: -P 2ac 2ab	Cell parameters from 3411 reflections
*a* = 20.045 (2) Å	θ = 2.5–20.5°
*b* = 13.2042 (19) Å	µ = 0.09 mm^−^^1^
*c* = 24.253 (3) Å	*T* = 298 K
*V* = 6419.2 (14) Å^3^	Block, light yellow
*Z* = 16	0.49 × 0.48 × 0.42 mm

### Data collection

**Table:**

Bruker SMART CCD diffractometer	5654 independent reflections
Radiation source: fine-focus sealed tube	2589 reflections with *I* > 2σ(*I*)
graphite	*R*_int_ = 0.092
φ and ω scans	θ_max_ = 25.0°, θ_min_ = 1.7°
Absorption correction: multi-scan (*SADABS*; Sheldrick, 1996)	*h* = −19→23
*T*_min_ = 0.958, *T*_max_ = 0.964	*k* = −15→15
25460 measured reflections	*l* = −28→22

### Refinement

**Table:**

Refinement on *F*^2^	Primary atom site location: structure-invariant direct methods
Least-squares matrix: full	Secondary atom site location: difference Fourier map
*R*[*F*^2^ > 2σ(*F*^2^)] = 0.063	Hydrogen site location: inferred from neighbouring sites
*wR*(*F*^2^) = 0.206	H-atom parameters constrained
*S* = 1.12	*w* = 1/[σ^2^(*F*_o_^2^) + 11.5471*P*] where *P* = (*F*_o_^2^ + 2*F*_c_^2^)/3
5654 reflections	(Δ/σ)_max_ = 0.001
405 parameters	Δρ_max_ = 0.21 e Å^−^^3^
0 restraints	Δρ_min_ = −0.22 e Å^−^^3^

### Special details

**Table:**

Geometry. All e.s.d.'s (except the e.s.d. in the dihedral angle between two l.s. planes) are estimated using the full covariance matrix. The cell e.s.d.'s are taken into account individually in the estimation of e.s.d.'s in distances, angles and torsion angles; correlations between e.s.d.'s in cell parameters are only used when they are defined by crystal symmetry. An approximate (isotropic) treatment of cell e.s.d.'s is used for estimating e.s.d.'s involving l.s. planes.
Refinement. Refinement of *F*^2^ against ALL reflections. The weighted *R*-factor *wR* and goodness of fit *S* are based on *F*^2^, conventional *R*-factors *R* are based on *F*, with *F* set to zero for negative *F*^2^. The threshold expression of *F*^2^ > σ(*F*^2^) is used only for calculating *R*-factors(gt) *etc*. and is not relevant to the choice of reflections for refinement. *R*-factors based on *F*^2^ are statistically about twice as large as those based on *F*, and *R*- factors based on ALL data will be even larger.

### Fractional atomic coordinates and isotropic or equivalent isotropic displacement parameters (Å^2^)

**Table:**

	*x*	*y*	*z*	*U*_iso_*/*U*_eq_	
N1	0.09178 (18)	0.5134 (3)	0.75088 (16)	0.0456 (10)	
N2	0.16453 (19)	1.0602 (3)	0.98602 (16)	0.0473 (10)	
O1	0.18197 (15)	0.3410 (2)	0.65385 (12)	0.0514 (9)	
O2	0.21163 (16)	0.1364 (2)	0.67521 (14)	0.0556 (9)	
O3	0.20521 (18)	0.0660 (3)	0.77778 (15)	0.0695 (11)	
O4	0.03160 (18)	0.9210 (3)	0.71702 (15)	0.0705 (11)	
H4	−0.0089	0.9295	0.7151	0.106*	
O5	0.05416 (18)	0.9042 (3)	0.89798 (14)	0.0650 (10)	
O6	0.01757 (18)	0.6985 (3)	0.91633 (14)	0.0644 (10)	
O7	0.03635 (17)	0.6176 (2)	1.01429 (15)	0.0601 (10)	
O8	0.20925 (18)	1.4749 (3)	0.99430 (17)	0.0764 (12)	
H8	0.2482	1.4885	0.9871	0.115*	
C1	0.1457 (2)	0.4734 (4)	0.73373 (18)	0.0447 (12)	
H1	0.1741	0.5109	0.7114	0.054*	
C2	0.1638 (2)	0.3693 (4)	0.74853 (18)	0.0397 (11)	
C3	0.1825 (2)	0.3028 (4)	0.70673 (18)	0.0394 (11)	
C4	0.1949 (2)	0.2018 (4)	0.71691 (19)	0.0456 (12)	
C5	0.1922 (2)	0.1671 (4)	0.7710 (2)	0.0484 (13)	
C6	0.1770 (2)	0.2326 (4)	0.8130 (2)	0.0564 (14)	
H6	0.1768	0.2089	0.8492	0.068*	
C7	0.1621 (2)	0.3331 (4)	0.80274 (19)	0.0498 (13)	
C8	0.2416 (3)	0.3325 (5)	0.6226 (2)	0.0737 (17)	
H8A	0.2794	0.3402	0.6466	0.111*	
H8B	0.2425	0.3843	0.5949	0.111*	
H8C	0.2433	0.2672	0.6053	0.111*	
C9	0.1551 (3)	0.0923 (5)	0.6491 (3)	0.0854 (19)	
H9A	0.1323	0.0492	0.6748	0.128*	
H9B	0.1693	0.0531	0.6179	0.128*	
H9C	0.1255	0.1450	0.6370	0.128*	
C10	0.1876 (4)	0.0206 (5)	0.8287 (3)	0.103 (2)	
H10A	0.2147	0.0485	0.8576	0.155*	
H10B	0.1947	−0.0512	0.8266	0.155*	
H10C	0.1414	0.0339	0.8364	0.155*	
C11	0.1440 (3)	0.4002 (5)	0.8507 (2)	0.0784 (19)	
H11A	0.0965	0.4002	0.8556	0.118*	
H11B	0.1590	0.4680	0.8436	0.118*	
H11C	0.1650	0.3753	0.8836	0.118*	
C12	0.0783 (2)	0.6174 (4)	0.73988 (19)	0.0427 (12)	
C13	0.1269 (2)	0.6905 (4)	0.7358 (2)	0.0564 (14)	
H13	0.1716	0.6720	0.7382	0.068*	
C14	0.1104 (3)	0.7909 (4)	0.7282 (2)	0.0598 (15)	
H14	0.1441	0.8392	0.7259	0.072*	
C15	0.0449 (3)	0.8208 (4)	0.7239 (2)	0.0525 (13)	
C16	−0.0032 (2)	0.7480 (4)	0.7281 (2)	0.0600 (15)	
H16	−0.0479	0.7663	0.7253	0.072*	
C17	0.0131 (2)	0.6478 (4)	0.7362 (2)	0.0565 (14)	
H17	−0.0207	0.6000	0.7393	0.068*	
C18	0.1073 (2)	1.0219 (4)	0.97645 (19)	0.0497 (13)	
H18	0.0744	1.0626	0.9611	0.060*	
C19	0.0916 (2)	0.9151 (3)	0.98894 (19)	0.0431 (12)	
C20	0.0615 (2)	0.8572 (4)	0.94810 (19)	0.0437 (12)	
C21	0.0456 (2)	0.7570 (4)	0.9569 (2)	0.0441 (12)	
C22	0.0562 (2)	0.7160 (4)	1.0086 (2)	0.0455 (12)	
C23	0.0853 (2)	0.7717 (4)	1.0497 (2)	0.0499 (13)	
H23	0.0921	0.7426	1.0842	0.060*	
C24	0.1049 (2)	0.8722 (4)	1.0404 (2)	0.0472 (12)	
C25	−0.0055 (3)	0.8951 (6)	0.8685 (3)	0.103 (2)	
H25A	−0.0030	0.8376	0.8444	0.154*	
H25B	−0.0126	0.9553	0.8471	0.154*	
H25C	−0.0419	0.8864	0.8938	0.154*	
C26	0.0659 (3)	0.6435 (5)	0.8855 (3)	0.100 (2)	
H26A	0.0944	0.6900	0.8663	0.150*	
H26B	0.0437	0.6005	0.8593	0.150*	
H26C	0.0921	0.6027	0.9100	0.150*	
C27	0.0461 (3)	0.5706 (4)	1.0661 (2)	0.0809 (19)	
H27A	0.0929	0.5695	1.0747	0.121*	
H27B	0.0294	0.5025	1.0648	0.121*	
H27C	0.0226	0.6079	1.0940	0.121*	
C28	0.1402 (3)	0.9258 (4)	1.0866 (2)	0.0735 (17)	
H28A	0.1876	0.9187	1.0820	0.110*	
H28B	0.1270	0.8966	1.1212	0.110*	
H28C	0.1286	0.9963	1.0861	0.110*	
C29	0.1736 (2)	1.1670 (3)	0.98294 (18)	0.0427 (12)	
C30	0.2359 (2)	1.2045 (4)	0.9702 (2)	0.0481 (13)	
H30	0.2698	1.1602	0.9601	0.058*	
C31	0.2483 (2)	1.3071 (4)	0.9724 (2)	0.0507 (13)	
H31	0.2901	1.3317	0.9625	0.061*	
C32	0.1995 (2)	1.3735 (4)	0.9891 (2)	0.0481 (12)	
C33	0.1365 (2)	1.3370 (4)	1.0002 (2)	0.0570 (14)	
H33	0.1025	1.3815	1.0096	0.068*	
C34	0.1242 (2)	1.2354 (4)	0.9973 (2)	0.0526 (13)	
H34	0.0816	1.2116	1.0052	0.063*	

### Atomic displacement parameters (Å^2^)

**Table:**

	*U*^11^	*U*^22^	*U*^33^	*U*^12^	*U*^13^	*U*^23^
N1	0.043 (2)	0.043 (3)	0.051 (2)	0.0044 (19)	−0.0004 (19)	−0.006 (2)
N2	0.047 (2)	0.040 (3)	0.055 (3)	−0.0077 (19)	0.004 (2)	−0.001 (2)
O1	0.054 (2)	0.058 (2)	0.042 (2)	0.0144 (17)	0.0005 (16)	0.0045 (17)
O2	0.061 (2)	0.049 (2)	0.057 (2)	0.0103 (18)	0.0036 (18)	−0.0065 (18)
O3	0.077 (3)	0.059 (3)	0.072 (3)	0.017 (2)	0.014 (2)	0.020 (2)
O4	0.068 (2)	0.040 (2)	0.103 (3)	0.0017 (18)	0.023 (2)	0.006 (2)
O5	0.073 (3)	0.070 (3)	0.052 (2)	−0.020 (2)	−0.0142 (19)	0.0144 (19)
O6	0.071 (3)	0.064 (3)	0.058 (2)	−0.011 (2)	−0.0055 (19)	−0.015 (2)
O7	0.073 (2)	0.038 (2)	0.069 (2)	−0.0106 (18)	−0.0024 (19)	0.0068 (19)
O8	0.069 (3)	0.038 (2)	0.122 (4)	−0.0053 (19)	0.000 (2)	−0.004 (2)
C1	0.045 (3)	0.047 (3)	0.042 (3)	0.005 (2)	−0.003 (2)	−0.002 (2)
C2	0.035 (2)	0.049 (3)	0.036 (3)	0.007 (2)	−0.002 (2)	0.000 (2)
C3	0.036 (3)	0.045 (3)	0.038 (3)	0.005 (2)	−0.003 (2)	0.002 (2)
C4	0.042 (3)	0.049 (3)	0.046 (3)	0.007 (2)	0.004 (2)	−0.001 (3)
C5	0.046 (3)	0.045 (3)	0.054 (3)	0.015 (2)	0.002 (2)	0.010 (3)
C6	0.057 (3)	0.069 (4)	0.043 (3)	0.014 (3)	0.003 (2)	0.016 (3)
C7	0.050 (3)	0.061 (4)	0.038 (3)	0.013 (3)	−0.001 (2)	−0.003 (3)
C8	0.067 (4)	0.097 (5)	0.057 (3)	0.013 (3)	0.016 (3)	0.017 (3)
C9	0.095 (5)	0.078 (5)	0.084 (5)	−0.012 (4)	−0.007 (4)	−0.029 (4)
C10	0.140 (6)	0.072 (5)	0.098 (5)	0.018 (4)	0.027 (5)	0.042 (4)
C11	0.096 (5)	0.096 (5)	0.043 (3)	0.034 (4)	−0.002 (3)	−0.006 (3)
C12	0.040 (3)	0.040 (3)	0.048 (3)	0.003 (2)	0.003 (2)	−0.005 (2)
C13	0.041 (3)	0.050 (4)	0.078 (4)	0.002 (3)	0.014 (3)	−0.007 (3)
C14	0.051 (3)	0.046 (3)	0.082 (4)	−0.008 (3)	0.017 (3)	−0.003 (3)
C15	0.053 (3)	0.039 (3)	0.066 (3)	0.003 (3)	0.013 (3)	0.001 (3)
C16	0.043 (3)	0.047 (3)	0.091 (4)	0.005 (3)	−0.001 (3)	0.002 (3)
C17	0.039 (3)	0.041 (3)	0.090 (4)	−0.001 (2)	−0.003 (3)	0.000 (3)
C18	0.051 (3)	0.045 (3)	0.054 (3)	−0.004 (2)	−0.002 (2)	0.002 (3)
C19	0.042 (3)	0.036 (3)	0.051 (3)	−0.003 (2)	0.000 (2)	−0.004 (2)
C20	0.044 (3)	0.046 (3)	0.041 (3)	−0.004 (2)	0.001 (2)	0.003 (2)
C21	0.042 (3)	0.042 (3)	0.048 (3)	−0.009 (2)	−0.004 (2)	−0.007 (3)
C22	0.043 (3)	0.039 (3)	0.054 (3)	−0.005 (2)	0.004 (2)	−0.003 (3)
C23	0.055 (3)	0.048 (3)	0.046 (3)	−0.004 (3)	−0.006 (2)	0.003 (3)
C24	0.052 (3)	0.043 (3)	0.046 (3)	−0.007 (2)	−0.005 (2)	−0.001 (3)
C25	0.099 (5)	0.119 (6)	0.090 (5)	−0.004 (4)	−0.043 (4)	0.030 (5)
C26	0.126 (6)	0.093 (5)	0.082 (5)	−0.013 (4)	0.028 (4)	−0.038 (4)
C27	0.113 (5)	0.049 (4)	0.081 (4)	−0.013 (3)	−0.001 (4)	0.019 (3)
C28	0.108 (5)	0.059 (4)	0.054 (3)	−0.024 (3)	−0.015 (3)	−0.002 (3)
C29	0.046 (3)	0.038 (3)	0.044 (3)	−0.005 (2)	0.002 (2)	−0.002 (2)
C30	0.043 (3)	0.040 (3)	0.061 (3)	−0.001 (2)	0.003 (2)	−0.002 (2)
C31	0.043 (3)	0.045 (3)	0.063 (3)	−0.010 (2)	0.000 (3)	0.003 (3)
C32	0.051 (3)	0.033 (3)	0.060 (3)	−0.004 (2)	−0.007 (3)	0.003 (2)
C33	0.050 (3)	0.043 (3)	0.078 (4)	0.003 (3)	0.005 (3)	−0.004 (3)
C34	0.041 (3)	0.048 (3)	0.068 (4)	−0.005 (2)	0.006 (2)	−0.001 (3)

### Geometric parameters (Å, °)

**Table:**

N1—C1	1.272 (5)	C12—C17	1.370 (6)
N1—C12	1.426 (6)	C12—C13	1.375 (6)
N2—C18	1.274 (5)	C13—C14	1.379 (7)
N2—C29	1.424 (6)	C13—H13	0.9300
O1—C3	1.378 (5)	C14—C15	1.375 (7)
O1—C8	1.420 (5)	C14—H14	0.9300
O2—C4	1.371 (5)	C15—C16	1.366 (6)
O2—C9	1.423 (6)	C16—C17	1.376 (7)
O3—C5	1.371 (6)	C16—H16	0.9300
O3—C10	1.417 (6)	C17—H17	0.9300
O4—C15	1.360 (5)	C18—C19	1.477 (6)
O4—H4	0.8200	C18—H18	0.9300
O5—C20	1.373 (5)	C19—C20	1.390 (6)
O5—C25	1.398 (6)	C19—C24	1.396 (6)
O6—C21	1.371 (5)	C20—C21	1.378 (6)
O6—C26	1.424 (6)	C21—C22	1.382 (6)
O7—C22	1.367 (5)	C22—C23	1.368 (6)
O7—C27	1.415 (6)	C23—C24	1.403 (6)
O8—C32	1.360 (5)	C23—H23	0.9300
O8—H8	0.8200	C24—C28	1.503 (6)
C1—C2	1.466 (6)	C25—H25A	0.9600
C1—H1	0.9300	C25—H25B	0.9600
C2—C3	1.392 (6)	C25—H25C	0.9600
C2—C7	1.399 (6)	C26—H26A	0.9600
C3—C4	1.380 (6)	C26—H26B	0.9600
C4—C5	1.389 (6)	C26—H26C	0.9600
C5—C6	1.371 (7)	C27—H27A	0.9600
C6—C7	1.383 (7)	C27—H27B	0.9600
C6—H6	0.9300	C27—H27C	0.9600
C7—C11	1.507 (7)	C28—H28A	0.9600
C8—H8A	0.9600	C28—H28B	0.9600
C8—H8B	0.9600	C28—H28C	0.9600
C8—H8C	0.9600	C29—C30	1.379 (6)
C9—H9A	0.9600	C29—C34	1.385 (6)
C9—H9B	0.9600	C30—C31	1.379 (6)
C9—H9C	0.9600	C30—H30	0.9300
C10—H10A	0.9600	C31—C32	1.375 (6)
C10—H10B	0.9600	C31—H31	0.9300
C10—H10C	0.9600	C32—C33	1.377 (6)
C11—H11A	0.9600	C33—C34	1.367 (7)
C11—H11B	0.9600	C33—H33	0.9300
C11—H11C	0.9600	C34—H34	0.9300
			
C1—N1—C12	120.0 (4)	C15—C16—H16	119.4
C18—N2—C29	119.8 (4)	C17—C16—H16	119.4
C3—O1—C8	117.5 (4)	C12—C17—C16	121.2 (5)
C4—O2—C9	113.0 (4)	C12—C17—H17	119.4
C5—O3—C10	118.0 (4)	C16—C17—H17	119.4
C15—O4—H4	109.5	N2—C18—C19	122.2 (5)
C20—O5—C25	120.4 (4)	N2—C18—H18	118.9
C21—O6—C26	112.7 (4)	C19—C18—H18	118.9
C22—O7—C27	117.8 (4)	C20—C19—C24	119.8 (4)
C32—O8—H8	109.5	C20—C19—C18	118.2 (4)
N1—C1—C2	121.3 (4)	C24—C19—C18	122.0 (4)
N1—C1—H1	119.4	O5—C20—C21	123.2 (4)
C2—C1—H1	119.4	O5—C20—C19	115.4 (4)
C3—C2—C7	118.4 (4)	C21—C20—C19	121.2 (4)
C3—C2—C1	118.6 (4)	O6—C21—C20	121.6 (4)
C7—C2—C1	123.0 (4)	O6—C21—C22	119.7 (4)
O1—C3—C4	121.4 (4)	C20—C21—C22	118.7 (4)
O1—C3—C2	116.4 (4)	O7—C22—C23	124.2 (5)
C4—C3—C2	121.9 (4)	O7—C22—C21	114.7 (4)
O2—C4—C3	121.4 (4)	C23—C22—C21	121.1 (4)
O2—C4—C5	119.9 (4)	C22—C23—C24	120.7 (5)
C3—C4—C5	118.7 (4)	C22—C23—H23	119.7
O3—C5—C6	124.5 (5)	C24—C23—H23	119.7
O3—C5—C4	115.3 (5)	C19—C24—C23	118.3 (4)
C6—C5—C4	120.3 (5)	C19—C24—C28	124.4 (4)
C5—C6—C7	121.2 (5)	C23—C24—C28	117.2 (4)
C5—C6—H6	119.4	O5—C25—H25A	109.5
C7—C6—H6	119.4	O5—C25—H25B	109.5
C6—C7—C2	119.5 (4)	H25A—C25—H25B	109.5
C6—C7—C11	118.5 (5)	O5—C25—H25C	109.5
C2—C7—C11	122.0 (5)	H25A—C25—H25C	109.5
O1—C8—H8A	109.5	H25B—C25—H25C	109.5
O1—C8—H8B	109.5	O6—C26—H26A	109.5
H8A—C8—H8B	109.5	O6—C26—H26B	109.5
O1—C8—H8C	109.5	H26A—C26—H26B	109.5
H8A—C8—H8C	109.5	O6—C26—H26C	109.5
H8B—C8—H8C	109.5	H26A—C26—H26C	109.5
O2—C9—H9A	109.5	H26B—C26—H26C	109.5
O2—C9—H9B	109.5	O7—C27—H27A	109.5
H9A—C9—H9B	109.5	O7—C27—H27B	109.5
O2—C9—H9C	109.5	H27A—C27—H27B	109.5
H9A—C9—H9C	109.5	O7—C27—H27C	109.5
H9B—C9—H9C	109.5	H27A—C27—H27C	109.5
O3—C10—H10A	109.5	H27B—C27—H27C	109.5
O3—C10—H10B	109.5	C24—C28—H28A	109.5
H10A—C10—H10B	109.5	C24—C28—H28B	109.5
O3—C10—H10C	109.5	H28A—C28—H28B	109.5
H10A—C10—H10C	109.5	C24—C28—H28C	109.5
H10B—C10—H10C	109.5	H28A—C28—H28C	109.5
C7—C11—H11A	109.5	H28B—C28—H28C	109.5
C7—C11—H11B	109.5	C30—C29—C34	118.0 (4)
H11A—C11—H11B	109.5	C30—C29—N2	118.9 (4)
C7—C11—H11C	109.5	C34—C29—N2	122.8 (4)
H11A—C11—H11C	109.5	C31—C30—C29	120.6 (5)
H11B—C11—H11C	109.5	C31—C30—H30	119.7
C17—C12—C13	117.7 (5)	C29—C30—H30	119.7
C17—C12—N1	118.4 (4)	C32—C31—C30	120.5 (5)
C13—C12—N1	123.8 (4)	C32—C31—H31	119.7
C12—C13—C14	121.0 (5)	C30—C31—H31	119.7
C12—C13—H13	119.5	O8—C32—C31	123.5 (5)
C14—C13—H13	119.5	O8—C32—C33	117.3 (5)
C15—C14—C13	121.0 (5)	C31—C32—C33	119.2 (5)
C15—C14—H14	119.5	C34—C33—C32	120.0 (5)
C13—C14—H14	119.5	C34—C33—H33	120.0
O4—C15—C16	123.8 (5)	C32—C33—H33	120.0
O4—C15—C14	118.4 (5)	C33—C34—C29	121.6 (5)
C16—C15—C14	117.8 (5)	C33—C34—H34	119.2
C15—C16—C17	121.2 (5)	C29—C34—H34	119.2
			
C12—N1—C1—C2	174.5 (4)	C29—N2—C18—C19	−168.3 (4)
N1—C1—C2—C3	129.8 (5)	N2—C18—C19—C20	−130.8 (5)
N1—C1—C2—C7	−49.3 (7)	N2—C18—C19—C24	50.4 (7)
C8—O1—C3—C4	−60.4 (6)	C25—O5—C20—C21	48.7 (7)
C8—O1—C3—C2	125.4 (5)	C25—O5—C20—C19	−136.6 (5)
C7—C2—C3—O1	178.6 (4)	C24—C19—C20—O5	−176.1 (4)
C1—C2—C3—O1	−0.5 (6)	C18—C19—C20—O5	5.0 (6)
C7—C2—C3—C4	4.4 (7)	C24—C19—C20—C21	−1.4 (7)
C1—C2—C3—C4	−174.6 (4)	C18—C19—C20—C21	179.8 (4)
C9—O2—C4—C3	−87.7 (6)	C26—O6—C21—C20	93.7 (6)
C9—O2—C4—C5	94.2 (5)	C26—O6—C21—C22	−88.7 (6)
O1—C3—C4—O2	4.4 (7)	O5—C20—C21—O6	−3.8 (7)
C2—C3—C4—O2	178.2 (4)	C19—C20—C21—O6	−178.2 (4)
O1—C3—C4—C5	−177.5 (4)	O5—C20—C21—C22	178.5 (4)
C2—C3—C4—C5	−3.6 (7)	C19—C20—C21—C22	4.1 (7)
C10—O3—C5—C6	15.0 (8)	C27—O7—C22—C23	0.4 (7)
C10—O3—C5—C4	−164.8 (5)	C27—O7—C22—C21	179.6 (4)
O2—C4—C5—O3	−1.7 (6)	O6—C21—C22—O7	−0.5 (6)
C3—C4—C5—O3	−179.9 (4)	C20—C21—C22—O7	177.2 (4)
O2—C4—C5—C6	178.5 (4)	O6—C21—C22—C23	178.7 (4)
C3—C4—C5—C6	0.3 (7)	C20—C21—C22—C23	−3.5 (7)
O3—C5—C6—C7	−177.7 (5)	O7—C22—C23—C24	179.3 (4)
C4—C5—C6—C7	2.1 (8)	C21—C22—C23—C24	0.1 (7)
C5—C6—C7—C2	−1.2 (7)	C20—C19—C24—C23	−2.0 (7)
C5—C6—C7—C11	178.3 (5)	C18—C19—C24—C23	176.8 (4)
C3—C2—C7—C6	−2.0 (7)	C20—C19—C24—C28	176.9 (5)
C1—C2—C7—C6	177.0 (4)	C18—C19—C24—C28	−4.3 (8)
C3—C2—C7—C11	178.6 (4)	C22—C23—C24—C19	2.7 (7)
C1—C2—C7—C11	−2.4 (7)	C22—C23—C24—C28	−176.4 (5)
C1—N1—C12—C17	152.0 (5)	C18—N2—C29—C30	−153.0 (5)
C1—N1—C12—C13	−31.9 (7)	C18—N2—C29—C34	33.4 (7)
C17—C12—C13—C14	−0.1 (8)	C34—C29—C30—C31	0.6 (7)
N1—C12—C13—C14	−176.3 (5)	N2—C29—C30—C31	−173.3 (4)
C12—C13—C14—C15	−0.6 (8)	C29—C30—C31—C32	2.2 (8)
C13—C14—C15—O4	179.3 (5)	C30—C31—C32—O8	177.2 (5)
C13—C14—C15—C16	0.7 (8)	C30—C31—C32—C33	−4.2 (8)
O4—C15—C16—C17	−178.6 (5)	O8—C32—C33—C34	−178.0 (5)
C14—C15—C16—C17	0.0 (8)	C31—C32—C33—C34	3.4 (8)
C13—C12—C17—C16	0.8 (8)	C32—C33—C34—C29	−0.5 (8)
N1—C12—C17—C16	177.2 (5)	C30—C29—C34—C33	−1.5 (7)
C15—C16—C17—C12	−0.7 (9)	N2—C29—C34—C33	172.2 (5)

### Hydrogen-bond geometry (Å, °)

**Table:**

*D*—H···*A*	*D*—H	H···*A*	*D*···*A*	*D*—H···*A*
O4—H4···N1^i^	0.82	2.16	2.866 (5)	144
O8—H8···N2^ii^	0.82	1.99	2.777 (5)	161

Symmetry codes: (i) −*x*, *y*+1/2, −*z*+3/2; (ii) −*x*+1/2, *y*+1/2, *z*.

## References

[bb1] Bruker (1997). *SMART* and *SAINT* Bruker AXS Inc., Madison, Wisconsin, USA.

[bb2] Sheldrick, G. M. (1996). *SADABS*, University of Göttingen, Germany.

[bb3] Sheldrick, G. M. (2008). *Acta Cryst.* A**64**, 112–122. 10.1107/S0108767307043930 18156677

[bb4] Spek, A. L. (2009). *Acta Cryst.* D**65**, 148–155. 10.1107/S090744490804362X PMC2631630 19171970

[bb5] Wang, C.-Y. (2009). *Acta Cryst.* E**65**, o56.

[bb6] Yu, T.-Z., Zhang, K., Yuling Zhao, Y.-L., Yang, C.-H., Zhang, H., Fan, D.-W. & Dong, W.-K. (2007). *Inorg. Chem. Commun* **10**, 401–403.

